# Antioxidant and Anti-Inflammatory Activities in Extracts from Minke Whale (*Balaenoptera acutorostrata*) Blubber

**DOI:** 10.1155/2017/3835851

**Published:** 2017-10-08

**Authors:** Mari Johannessen Walquist, Svein Kristian Stormo, Ida-Johanne Jensen, Bjarne Østerud, Karl-Erik Eilertsen

**Affiliations:** ^1^Norwegian College of Fishery Science, Faculty of Biosciences, Fisheries and Economics, UiT-The Arctic University of Norway, 9037 Tromsø, Norway; ^2^Nofima, Muninbakken 9-13, Pb 6122, 9291 Tromsø, Norway; ^3^Faculty of Health Sciences, IMB, K.G. Jebsen TREC, UiT-The Arctic University of Norway, 9037 Tromsø, Norway

## Abstract

Intake of long-chain omega-3 polyunsaturated fatty acids (LC-n3-PUFA) is commonly recognized to reduce cardiovascular disease (CVD). In previous studies, cold-pressed whale oil (CWO) and cod liver oil (CLO) were given as a dietary supplement to healthy volunteers. Even though CWO contains less than half the amount of LC-n3-PUFA of CLO, CWO supplement resulted in beneficial effects on anti-inflammatory and CVD risk markers compared to CLO. In the present study, we prepared virtually lipid-free extracts from CWO and CLO and evaluated the antioxidative capacity (AOC) and anti-inflammatory effects. Oxygen radical absorbance capacity (ORAC) and ferric reducing antioxidant power (FRAP) assays were used to test the AOC, and the results indicated high levels of antioxidants present in all extracts. The anti-inflammatory effects of the extracts were tested with lipopolysaccharide- (LPS-) treated THP-1 cells, measuring its ability to reduce cytokine and chemokine secretion. Several CWO extracts displayed anti-inflammatory activity, and a butyl alcohol extract of CWO most effectively reduced TNF-*α* (50%, *p* < 0.05) and MCP-1 (85%, *p* < 0.001) secretion. This extract maintained a stable effect of reducing MCP-1 secretion (60%, *p* < 0.05) even after long-term storage. In conclusion, CWO has antioxidant and anti-inflammatory activities that may act in addition to its well-known LC-n3-PUFA effects.

## 1. Introduction

Atherosclerosis is a cardiovascular disease (CVD) characterized by lipid accumulation and chronic inflammation in the arteries. The atheromatous plaques accumulate over years within the intima of arteries and may ultimately rupture, resulting in atherothrombosis and myocardial infarction [1]. Even though the mortality rate from CVD has decreased in high-income countries during the last decades, CVD remains the leading cause of mortality worldwide [2]. Thus, novel therapies to reduce the atherosclerotic risk and to prevent severe adverse effects associated with prevailing treatments are still needed. In recent years, the inflammatory aspects of atherosclerosis have been thoroughly elucidated [3–5]. Several cytokines and chemokines, such as tumor necrosis factor-alpha (TNF-*α*), interleukin-1*β* (IL-1*β*), interleukin-6 (IL-6), and monocyte chemoattractant protein-1 (MCP-1), are important contributors in atherogenesis and atherosclerosis [6–10]. Consequently, novel anti-inflammatory components may contribute significantly in both prevention and therapeutic treatment of atherosclerosis. Intake of long-chain omega-3 polyunsaturated fatty acids (LC-n3-PUFA) is a recognized risk reducer of CVD due to the triacylglycerol-lowering and anti-inflammatory effects [11–13]. As LC-n3-PUFA is very susceptible to oxidation, it seems important to eat sufficient amounts of antioxidants to prevent lipid peroxidation [14]. Thus, combining LC-n3-PUFA with other anti-inflammatory agents may be an effective approach to reduce atherosclerosis. Bioprospecting for antioxidants and anti-inflammatory components has led to an extended search in a wide range of marine species, mainly focusing on small organisms such as bacteria, fungi, and invertebrates [15]. In this context, larger marine mammals have received less attention. The minke whale (*Balaenoptera acutorostrata*) is an Atlantic finback whale regularly migrating to areas in the north where they feed on pelagic fish and crustaceans [16]. Minke whales have a thick layer of blubber [17] which is a vascularized hypodermal adipose tissue, vital for buoyancy, thermal insulation, and energy storage [18]. The blubber is a modified adipose tissue composed of adipocytes and connective tissue comprised of highly organized elastin and collagen fibers [19]. Intact blubber and oil extracted from blubber have been used in the diet in Arctic and Subarctic regions for centuries.

Previous dietary studies of cold-pressed oil from minke whale blubber (CWO) have indicated beneficial effects on CVD markers and improved an anti-inflammatory effect, also in comparison to cod liver oil (CLO) supplementation [20, 21]. Notably, the LC-n3-PUFA content in CWO was less than half when compared to CLO (10.3% versus 25.1%), indicating that the anti-inflammatory effects may rely on putative unknown components from blubber interacting with LC-n3-PUFA [21]. The objective in the present study was hence to elucidate possible *in vitro* antioxidative and anti-inflammatory effects of lipid-free extracts from CWO using biochemical assays and lipopolysaccharide- (LPS-) stimulated THP-1 cells.

## 2. Materials and Methods

### 2.1. Samples

Frozen fresh blubber from the ventral groove of the minke whale was provided from Ellingsen Seafood AS (Skrova, Norway).

### 2.2. Extraction

The blubber from the minke whale was grinded once before centrifugation at <2000 ×g (<40°C), and the oily top layer was collected (CWO-1). The remnant blubber was centrifuged again at the same speed, and again the oily top layer was collected (CWO-2). For comparison, commercially available CLO [22], rich in LC-n3-PUFA, was included. Each sample was further treated equally; 250 grams was extracted in 800 ml methanol/dichloromethane (1 : 1). The dichloromethane fraction containing most of the lipids was discarded, and residual dichloromethane in the methanol phase was removed by the use of a rotavapor. The remaining oil was removed by 3 × 200 ml heptane liquid-liquid extraction, and the methanol fraction was evaporated to almost dryness prior to addition of 100 ml water (dH_2_O). Further evaporation removed residual methanol from the extract. After evaporation to almost dryness, the extract was once again redissolved in 100 ml dH_2_O. The sample, now dissolved in water, was partitioned first with 3 × 200 ml ethyl acetate (EtOAc), then with 3 × 200 ml butyl alcohol (BuOH). The three extracts (EtOAc, BuOH, and H_2_O) were subsequently evaporated using a rotavapor and, finally, to dryness under a steam of nitrogen. Extracts were stored at −20°C prior to further analyses, and stock solutions (10 mg/ml) were prepared in dH_2_O and 5% dimethyl sulfoxide (DMSO).

### 2.3. Oxygen Radical Absorbance Capacity (ORAC)

The antioxidative effect was measured with the ORAC assay [23]. Samples were mixed with the synthetic free radical generator 2,2′-azobis-2-methyl-propanimidamide dihydrochloride (AAPH, Sigma-Aldrich) and the oxidizable fluorescein sodium salt (number F6377, Sigma-Aldrich) at physiological pH. The water-soluble vitamin E equivalent Trolox was used as standard, and fluorescence decay, as a result of the radical attack, was measured at 485 and 520 nm (Spectramax Gemini EM fluorimeter, Molecular Devices, Sunnyvale, USA). The antioxidative capacity (AOC) was defined as the net difference between the areas under the fluorescence decay curves for the sample and blank, respectively. The results are presented as *μ*mol Trolox equivalents (TE)/100 g oil. Extracts were measured at a concentration of 0.1 mg/ml sample.

### 2.4. Ferric Reducing Antioxidant Power (FRAP)

The antioxidant activities of the extracts were also evaluated using FRAP assay [24]. Samples were incubated together with the FRAP reagents (ferric-tripyridyltriazine complex, pH 3.6) in a microtiter plate for 30 minutes. The intense blue color formed as a result of reduction to the ferrous-tripyridyltriazine complex was measured at 593 nm (Spectramax Gemini EM fluorimeter, Molecular Devices, Sunnyvale, USA). The AOC was determined as TE from the Trolox standard curve (0–1000 *μ*M). The results are presented as *μ*mol TE/100 g oil.

### 2.5. Differentiated THP-1 Cells

THP-1 (number TIB-202, ATCC) is a monocyte cell line derived from a patient with acute monocytic leukemia. The cell line grows in suspension; however, after treatment with phorbol 12-myristate 13-acetate (PMA), the cells differentiate into adherent macrophage-like cells [25]. The cells were maintained in RPMI-1640 (FG1385, Biochrom) media with 10% fetal bovine serum (FBS) (S0115, Biochrome) and 10 *μ*g/ml gentamicin (A2712, Biochrome). Cells were incubated in 5% CO_2_ atmosphere and 37°C for all THP-1-based experiments and subcultured every 3-4 days when cell concentration reached 8 × 10^5^ viable cells. Every batch of THP-1 cells was evaluated with dose response against of LPS prior to use.

### 2.6. Anti-Inflammatory Screening Assay

Approximately 1 × 10^5^ THP-1 cells were seeded out and differentiated with 50 ng/ml PMA (P1585, Sigma-Aldrich) in 96-well plates. After 48 hours of incubation, cells were washed with PBS and fresh RPMI (without PMA) was added. The plates were incubated for 24 hours before the addition of 90 *μ*l fresh RPMI and 10 *μ*l extracts in different concentrations (50 *μ*g/ml, 10 *μ*g/ml, and 1 *μ*g/ml) to the respective wells. After a 1-hour incubation, LPS (L2630, Sigma-Aldrich) was added at a final concentration of 5 ng/ml to all wells except for the negative controls. The plates were then incubated for 6 hours and immediately frozen at −80°C. Negative, positive, and DMSO controls (0.05%) were included in every run.

### 2.7. ELISA

#### 2.7.1. Tumor Necrosis Factor-Alpha (TNF-*α*)

One day prior to the ELISA testing of TNF-*α* secretion, MaxiSorp 96F-well plates (Nunc) were coated with 2 *μ*g/ml capture antibody (eBioscience Inc., San Diego, CA, USA) and stored at 4°C, overnight. All incubations were at room temperature with shaking, and plates were washed with Tris-buffered saline (TBS) (pH 7.4, 0.05% Tween-20) between each step. Two hundred *μ*l blocking buffer was added to each well before a 1-hour incubation. TNF-*α* samples were diluted at 1 : 4 and 1 : 10; TNF-*α* was added to each well before 2 hours of incubation. Biotin coupled anti-human antibody (eBioscience Inc., San Diego, CA, USA) was diluted in TBS + 1% bovine serum albumin (BSA) to 3 *μ*g/ml and added to each well and subsequently incubated for 1 hour. Diluted ExtrAvidin®-Alkaline Phosphatase (Sigma-Aldrich) was added to each well prior to 30 min incubation. Finally, 100 *μ*l pNPP substrate (Sigma-Aldrich, 1 mg/ml in 1 M buffer, pH 9.8) was added to each well and incubated for 45 min before the plates were read at 405 nm.

#### 2.7.2. Monocyte Chemoattractant Protein-1 (MCP-1)

MCP-1 secretion was analyzed with a quantitative sandwich enzyme-linked immunosorbent assay (Human CCL2, MCP-1, ELISA kit, 88-7399, eBioscience Inc., San Diego, CA, USA) according to the manufacturer's protocol.

### 2.8. Cell Viability

The cell viability was measured with the thiazolyl blue tetrazolium bromide (MTT) assay [26] in HT29 cells (HTB-38, ATCC). Approximately 1 × 10^5^ cells were seeded in 96-well plates. After 48 hours of incubation, cells were washed with PBS before fresh media was added. To each well, 100 *μ*l of extracts (final concentration of 50 *μ*g), cell control, or deoxycholic acid (DCA, 500 *μ*M as a positive control) was added. The plates were incubated for 24 hours, washed with PBS, and then 15 *μ*l MTT (M2128, Sigma-Aldrich) was added to each well prior to 4 hours of incubating. One hundred *μ*l of solubilizing buffer (2-propanol, hydrogen chloride, and Triton-x-100) was finally added to each well, and the plates were incubated for 4 hours before read at 570 nm.

### 2.9. Bio-Plex^®^ Multiplex System (Bio Rad)

This system enables detection and quantification of multiple analyses in a single sample volume. Three customized express plates (6-plex, 96-well flat bottom) preblended from human cytokine group 1 (TNF-*α*, MCP-1, IL-6, interleukin-10 (10), interferon gamma (IFN-γ), and RANTES) were used in this study. The assay was performed according to the manufacturer's protocol (Bio-Rad Laboratories Inc., Hercules, California, USA). These assays were performed after long-term storage (>4 years at −20°C) of the extracts to investigate whether the anti-inflammatory effects were stable through storage.

### 2.10. Thin-Layer Chromatography (TLC)

Thin-layer chromatography was used to classify lipid classes. One *μ*l (25 mg/ml in dichloromethane (DCM)) of the oil samples and 5 *μ*l (1 mg/ml in DCM) extracts were analyzed on HP-TLC silica 60 plates (Merck Millipore, Billerica, Massachusetts, USA) in a solvent system of heptane:diethyl ether:acetic acid (70 : 30 : 1 *v*/*v*/*v*) to separate sample lipids. The plates were then treated with copper solution (10% cupric sulfate in 8% phosphoric acid), air dried for 10 minutes, and finally put in a cold oven and heated to 170°C. The standards used were 16-1A and 18-5A (Nu-Checkprep, Elysian, USA).

### 2.11. Statistical Methods

All of the statistical analyses were performed using IBM SPSS Statistics for Macintosh (released 22.0.0.0, SPSS Inc., Chicago, IL, USA). Values are presented as mean ± SD unless otherwise stated. The significance of differences was evaluated using one-way ANOVA, with Tukey's post hoc test. The difference at the level of *p* < 0.05 was considered statistically significant.

## 3. Results

### 3.1. Extraction Yield

**Figure 1:**
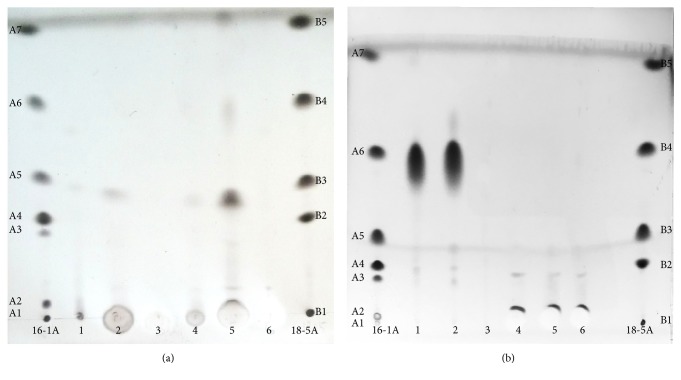
HP-TLC of oils (prior to extraction) and extracts. Fatty acid standards: 16-1A containing phospholipids (A1), monoacylglycerol (A2), diacylglycerol (A3), cholesterol (A4), free fatty acids (A5), triacylglycerol (A6), and cholesteryl ester (A7). 18-5A containing phospholipids (B1), cholesterol (B2), free fatty acids (B3), triacylglycerol (B4), and cholesteryl ester (B5). (a) Extracts: CWO-1 EtOAc (1), CWO-1 BuOH (2), CWO-1 H_2_O (3), CWO-2 EtOAc (4), CWO-2 BuOH (5), and CWO-2 H_2_O (6). (b) Oils before extraction: CLO (1) and CWO (2) empty (3) extracts: CLO-1 H_2_O (4), CLO-1 BuOH (5), and CLO EtOAc (6). HP-TLC = high performance thin-layer chromatography; CWO = cold-pressed whale oil; CLO = cod liver oil; EtOAc = ethyl acetate; BuOH = butyl alcohol.

Cold-pressed whale oil was separated into two consecutive samples (CWO-1 and CWO-2) after repeated grinding and centrifugation of the whale blubber. These samples were together with CLO further fractionated into three extracts (EtOAc, BuOH, and H_2_O) each. There were large differences in extraction yields (expressed as dry matter yields produced from 250 g of the respective oils; mg/g). Dry weights of the extracts from CWO-1 were 0.33 mg/g (EtOAc), 2.07 mg/g (BuOH), and 1.58 mg/g (H_2_O), and for the CWO-2 extracts, yields were 0.43 mg/g (EtOAc), 0.57 mg/g (BuOH), and 2.17 mg/g (H_2_O). Contrary to the CWO extracts, the CLO extracts contained only small amounts of water-soluble polar components as reflected in the dry matter yields of 0.60 mg/g (EtOAc), 0.054 mg/g (BuOH), and 0.008 mg/g (H_2_O). TLC (Figures 1(a) and 1(b)) confirmed that all of the extracts prepared from both CWO and CLO contained very few remaining lipids. It was also evident that the BuOH extracts from CWO-1 and CWO-2 contained very low amounts of residual free fatty acids and no triacylglycerol (TAG), diacylglycerol (DAG), or monoacylglycerol (MAG) (Figure 1(a), lanes 1–6). EtOAc extracts (from CWO-1 and CWO-2) contained some residual phospholipids, whereas the H_2_O extracts contained no lipids. All the extracts from CLO (EtOAc, BuOH, and H_2_O; Figure 1(b), lanes 4–6) contained trace amounts of lipids in the form of MAG and DAG, but no TAG. The whale blubber oil and cod liver oil both contained almost exclusively TAG (Figure 1(b), lanes 1 and 2). It is noteworthy that five times more extracts than oils were applied onto the TLC plates.

### 3.2. Antioxidative Capacity

**Figure 2:**
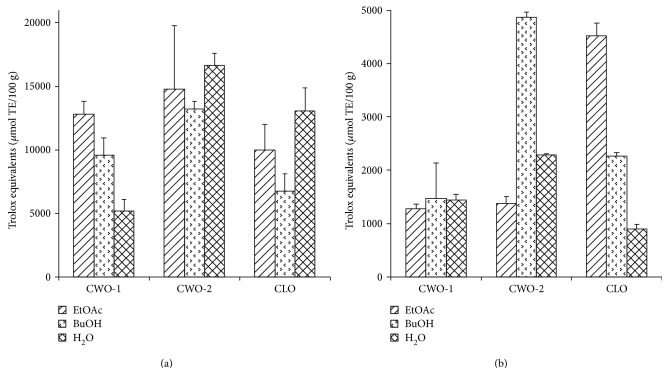
Antioxidative capacity in the extracts. CWO-1, CWO-2, and CLO which were sequentially extracted using EtOAc, BuOH, and water. The results are shown as Trolox equivalents (*μ*mol TE/100 g). (a) ORAC assay with extract concentrations of 0.1 mg/ml. (b) FRAP assay with extract concentrations of 10 mg/ml. CWO = cold-pressed whale oil; CLO = cod liver oil; EtOAc = ethyl acetate; BuOH = butyl alcohol; ORAC = oxygen radical absorbance capacity; FRAP = ferric reducing antioxidant power.

Two methods, ORAC (Figure 2(a)) and FRAP (Figure 2(b)), were applied to determine the AOC of the extracts. In the ORAC assay, it was evident that CWO-2 had high AOC in all three extracts (CWO-2-EtOAc, CWO-2-BuOH, and CWO-2-H_2_O extracts), and correspondingly, CWO-2 had the highest total AOC. In the FRAP assay, on the other hand, CWO-2-BuOH together with CLO-EtOAc displayed the highest AOC.

### 3.3. Anti-Inflammatory Effect on Cytokine Secretion in LPS-Treated THP-1 Cells

**Figure 3:**
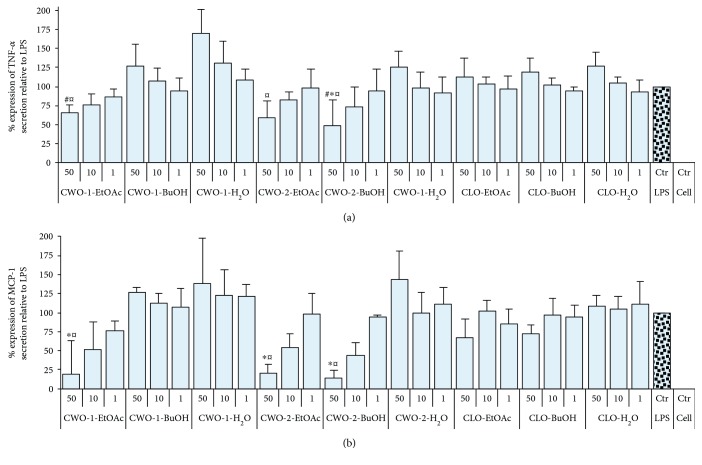
ELISA results for relative response of TNF-*α* and MCP-1 in LPS-treated THP-1 cells. Each extract was tested at three different concentrations (50 *μ*g/ml, 10 *μ*g/ml, and 1 *μ*g/ml), and the results are presented as mean with SD displayed as positive bars (*n* = 3). (a) TNF-*α* secretion relative to control. ^∗^*p* < 0.05 compared to LPS control. ^#^*p* < 0.05 compared to CLO-BuOH 50 *μ*g/ml. ^¤^*p* < 0.05 compared to CLO-H_2_O 50 *μ*g/ml. (b) MCP-1 secretion relative to control. ^∗^*p* < 0.001 compared to LPS control. ^¤^*p* < 0.001 compared to CLO-H_2_O 50 *μ*g/ml. TNF-*α* = tumor necrosis factor-alpha; MCP-1 = monocyte chemoattractant protein-1; LPS = lipopolysaccharide; CWO = cold-pressed whale oil; CLO = cod liver oil; EtOAc = ethyl acetate; BuOH = butyl alcohol.

**Figure 4:**
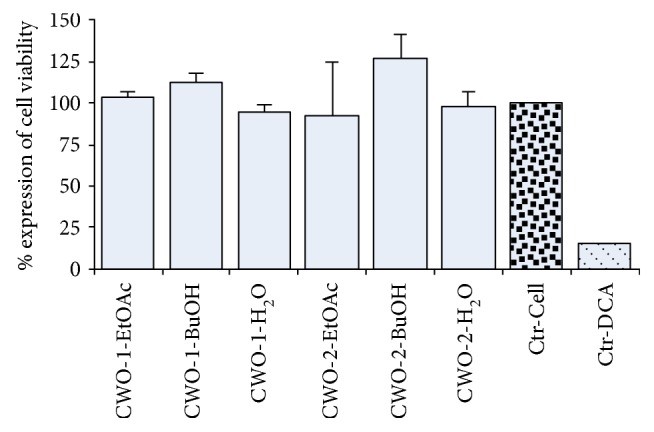
MTT results for cell viability of CWO-1 and CWO-2 extracts. Each extract was tested at 50 *μ*g/ml, and the results are presented as mean with SD displayed as positive bars (*n* = 3). The cell viability was calculated relative to the cell control (cell media), and 500 *μ*M DCA was included as a positive control. MTT = thiazolyl blue tetrazolium bromide; CWO = cold-pressed whale oil; EtOAc = ethyl acetate; BuOH = butyl alcohol; DCA = deoxycholic acid.

The anti-inflammatory effects of the extracts were studied using LPS-stimulated THP-1 macrophage-like cells. Inhibition of LPS-induced TNF-*α* and MCP-1 production (Figures 3(a) and 3(b), resp.) was most pronounced for CWO-2-BuOH. The extracts were applied at 3 different concentrations, and a dose-dependent inhibition of TNF-*α* and MCP-1 secretion was observed for this extract. When applying a dose of 50 *μ*g/ml (CWO-2-BuOH), TNF-*α* and MCP-1 production were reduced by 50% (*p* < 0.05) and 85% (*p* < 0.001), respectively. The extracts CWO-1-EtOAc and CWO-2-EtOAc also inhibited MCP-1 secretion dose dependently, and with a final extract concentration of 50 *μ*g/ml, the MCP-1 secretion was reduced by approximately 80% (*p* < 0.001, Figure 3(b)). A nonsignificant tendency of the reduction of TNF-*α* secretion was also observed for CWO-1-EtOAc and CWO-2-EtOAc (Figure 3(a)). None of the extracts affected LPS-induced IL-1*β* production (results not shown). Negative controls with cell media or vehicle (0.05% DMSO) did not stimulate the release of any of the tested cytokines. Cells incubated with extracts made from CLO were also analyzed; however, no effects were observed on TNF-*α* (Figure 3(a)), MCP-1 (Figure 3(b)), or IL-1*β* secretion (results not shown). When comparing the effects of treatment of THP-1 cells with all the extracts from CWO-1 and CWO-2 with the corresponding extracts from CLO, lower LPS-induced TNF-*α* secretion was observed for CWO-1-EtOAc (*p* < 0.05) and CWO-2-BuOH (*p* < 0.005) relative to treatment with CLO-BuOH (50 *μ*g/ml for all extracts, Figure 3(a)). The levels of LPS-induced TNF-*α* release (*p* < 0.05) after treatment of the THP-1 cells with 50 *μ*g/ml of CWO-1-EtOAc, CWO-2-EtOAc, or CWO-2-BuOH were lower compared to LPS-induced TNF-*α* production after treatment with 50 *μ*g/ml CLO-H_2_O (Figure 3(a)). There was a similar pattern for MCP-1, as LPS-induced MCP-1 release from THP-1 cells treated with extracts (at 50 *μ*g/ml) from CWO-1-EtOAc, CWO-2-EtOAc, or CWO-2-BuOH was different (*p* < 0.001) compared to treatment with 50 *μ*g/ml of CLO-H_2_O extract. The cell viability was tested using the MTT assay, and none of the CWO-1 or CWO-2 extracts affected the cell viability (Figure 4()).

### 3.4. Effect of Storage on Anti-Inflammatory Activity of the Extracts

The effects of the extracts after long-term storage (>4 years at −20°C) on LPS-induced production of TNF-*α*, MCP-1, IL-6, IL-10, and RANTES were also assessed (Table 1). CWO-2-BuOH (50 *μ*g/ml) inhibited MCP-1 production in LPS-treated THP-1 cells with more than 60% (*p* < 0.05). In cells treated with CWO-1-EtOAc, both LPS-induced TNF-*α* and MCP-1 were reduced, whereas secretion of anti-inflammatory IL-10 was increased in cells treated with CWO-2-EtOAc (*p* = 0.054, Table 1). Secretion of LPS-induced RANTES was not affected by treatment with any of the extracts (Table 1). CWO-2-BuOH (50 *μ*g/ml) inhibited IL-6 production (*p* = 0.058) compared to CLO-BuOH (50 *μ*g/ml), and CWO-2-EtOAc (50 *μ*g/ml) increased IL-10 production (*p* = 0.077) compared to CLO-EtOAc (50 *μ*g/ml, Table 1).

## 4. Discussion

Previous intervention studies comparing intake of CWO with intake of CLO indicated that CWO has anti-inflammatory effects not observed after intake of CLO [20, 21]. Epidemiological studies during the 1970s indicated anti-inflammatory and antioxidative effects of ingestion of blubber based on the low incidence of CVD among the indigenous people in Greenland [27, 28]. It is however important to notice that the Inuit consumed mainly fish in addition to meat and blubber from seals and whales. It has also been claimed that the prevalence of CVD was underestimated in this population [29, 30].

In our study, most of the lipids, and thus the fatty acids, were removed prior to *in vitro* testing to investigate whether blubber contained compounds that could act in synergy or in addition to the anti-inflammatory effects previously ascribed to the LC-n3-PUFA. To prevent the destruction of putative temperature-labile lipophilic antioxidants present in the whale blubber, extracts were prepared from CWO at low temperatures (<40°C). After removal of the most lipophilic parts of the oil samples, extracts with different polarity were prepared using EtOAc, BuOH, and water as extraction solvents. Thin-layer chromatography indicated that the BuOH extracts from CWO samples contained very low amounts of residual free fatty acids and trace amounts of TAG, DAG, or MAG. The CWO-1-EtOAc and CWO-2-EtOAc extracts contained some residual lipids, whereas the H_2_O extracts contained no lipids. For the extracts prepared from CLO, the TLC analysis showed that all these extracts contained very low amounts of free fatty acids, TAG, DAG, and MAG.

The total AOC observed in the extracts in this work may be considered high compared to other organic materials tested with FRAP and ORAC assays [31, 32]. The high AOC indicates that the tested extracts contain antioxidants that may protect against reactive oxygen species *in vivo*. ORAC assay is regarded more physiologically relevant than FRAP due to pH and temperature; however, it is important to emphasize that both these assays are simplified methods to measure AOC. These assays measure quite different mechanisms and are not fully comparable, and it was not surprising that the CWO-2-BuOH displayed high reducing power in the FRAP assay and lower ORAC activity. However, the total AOC from each sample shows the same ranking order (CWO-2 > CLO > CWO-1). Another important aspect is the dry matter yield. Being 5-6 times lower in CLO compared to CWO-2 and CWO-1, this results in lower total amount of antioxidants in CLO. Despite the simplicity of the assays used, these results provided the fundament for the investigation of anti-inflammatory effects.

Several of the extracts displayed potent and dose-dependent anti-inflammatory activity demonstrated through the reduction of LPS-induced production of chemokine (MCP-1) and cytokine (TNF-*α*). The most pronounced inhibition of TNF-*α* and MCP-1 was observed in cells treated with the CWO-2-BuOH extract, but treatment with CWO-1-EtOH and CWO-2-EtOAc also inhibited TNF-*α* production. It is possible that the concentrations (50, 10, and 1 *μ*g/ml) used in the present study might have been too low to reveal the full anti-inflammatory potential of the extracts.

All the extracts that inhibited LPS-induced TNF-*α* also inhibited release of MCP-1. However, the inhibiting effects on LPS-induced MCP-1 were much more pronounced, as MCP-1 levels were reduced 80% to 85% by these extracts. MCP-1 is an important contributor for atherosclerosis, and therefore potent and specific inhibitors of MCP-1 may be attractive drug candidates for the prevention of atherosclerosis. Contrary to the extracts produced from CWO, none of the CLO extracts affected any of the investigated cytokines or chemokines. The levels of secreted IL-1*β* were unchanged after treatment with all the extracts, apparently due to the different regulation mechanisms for IL-1*β* compared to the other cytokines and chemokines tested in this study [33].

The nature of many bioprospecting projects involves sample collection at very remote locations, before processing, extracting, and further investigation at different laboratories. This actualized a desire to determine if the putative bioactive compounds were sufficiently robust to be discovered with this kind of approach. The effects of long-term storage (>4 years at −20°C) on the anti-inflammatory activity in our extracts were investigated with a multiplex assay including MCP-1, TNF-*α*, IL-10, IL-6, RANTES, and IFN-*γ*. The anti-inflammatory activity was preserved for CWO-2-BuOH reducing MCP-1 secretion compared to LPS control. Interestingly, CWO-2-EtOAc increased the IL-10 production compared to both LPS control and CLO-EtOAc. IL-10 is considered to have anti-inflammatory capacity *in vivo* and is known to hold a critical role as a feedback regulator of a wide range of immune responses [34]. Secreted RANTES levels were not affected by any of the extracts, while CWO-2-BuOH downregulated the MCP-1 secretion. Different signaling pathways activate the production of RANTES and MCP-1 after LPS/Toll-like receptor 4 stimulation. RANTES belongs to the MyD88-dependent pathway [35, 36] whereas MCP-1 belongs to the MyD88-independent pathway [37]. In this study, virtually all of the hydrophobic heptan-dissolvable lipids were removed during the extraction process to establish anti-inflammatory activities independent of LC-n3-PUFA. Since triacylglycerol has also been found to downregulate MCP-1 expression in THP-1 cells [38], additional effects could be expected if the fatty acids were included in the extracts. During the refining process of commercial CLO, the oils are subjected to high temperature treatment, which may cause degradation and loss of putative anti-inflammatory compounds. This might explain the absence of anti-inflammatory activity in the CLO extracts despite the presence of antioxidative capacities.

## 5. Conclusion

This study has demonstrated that CWO contains antioxidants and anti-inflammatory activities that are not related to its content of LC-n3-PUFA. This indicates that there are unidentified extractable anti-inflammatory compounds present in whale oil yet to be discovered. To investigate the anti-inflammatory effects further, the putative bioactive components need to be isolated and CWO (still containing the fatty acids) should be evaluated *in vivo* in chronic inflammatory animal models.

## Figures and Tables

**Table 1:** Cytokine responses in LPS-induced THP-1 cells treated with different extracts result in relative % compared to LPS control. ^∗^*p* < 0.05 compared to LPS control, ^∗∗^*p* = 0.054 compared to LPS control and *p* = 0.077 compared to CLO-EtOAc 50 *μ*g/ml, and ^#^*p* = 0.058 compared to CLO-BuOH 50 *μ*g/ml. TNF-*α*: tumor necrosis factor-alpha; MCP-1: monocyte chemoattractant protein-1; IL-6: interleukin-6; IL-10: interleukin-10; RANTES: regulated on activation, normal T cell expressed, and secreted; LPS: lipopolysaccharide; CWO: cold-pressed whale oil; CLO: cod liver oil; EtOAc: ethyl acetate; BuOH: butyl alcohol.

Extracts	Extract conc. (*μ*g/ml)	IL-6 (% of LPS)	IL-10 (% of LPS)	MCP-1 (% of LPS)	RANTES (% of LPS)	TNF-*α* (% of LPS)
CWO-1-H_2_O	50	282 ± 92	212 ± 208	97 ± 17	126 ± 26	136 ± 81
	10	163 ± 40	92 ± 29	125 ± 23	141 ± 8	142 ± 67
	1	120 ± 20	99 ± 115	115 ± 18	128 ± 6	111 ± 38
CWO-1-BuOH	50	117 ± 3	102 ± 29	91 ± 2	110 ± 26	92 ± 37
	10	60 ± 53	ND	68 ± 56	91 ± 73	78 ± 65
	1	95 ± 17	92 ± 21	103 ± 15	116 ± 2	102 ± 39
CWO-1-EtOAc	50	85 ± 9	219 ± 106	58 ± 7	101 ± 27	60 ± 14
	10	90 ± 3	99 ± 35	100 ± 8	115 ± 10	90 ± 30
	1	106 ± 19	115 ± 25	117 ± 23	117 ± 12	104 ± 41
CWO-2-H_2_O	50	170 ± 49	166 ± 121	100 ± 11	109 ± 22	114 ± 50
	10	96 ± 7	91 ± 22	104 ± 9	127 ± 9	104 ± 22
	1	99 ± 16	95 ± 19	102 ± 13	137 ± 21	102 ± 36
CWO-2-BuOH	50	21 ± 12^#^	160 ± 43	37 ± 13^∗^	80 ± 25	42 ± 18
	10	71 ± 13	123 ± 35	105 ± 11	126 ± 24	91 ± 33
	1	91 ± 31	ND	108 ± 35	112 ± 32	103 ± 57
CWO-2-EtOAc	50	105 ± 34	272 ± 133^∗∗^	53 ± 11	98 ± 28	101 ± 65
	10	95 ± 17	137 ± 68	96 ± 12	141 ± 45	96 ± 38
	1	106 ± 16	112 ± 19	110 ± 12	135 ± 22	107 ± 42
CLO-H_2_O	50	141 ± 47	145 ± 99	91 ± 10	99 ± 11	89 ± 36
	10	112 ± 38	ND	99 ± 18	115 ± 25	97 ± 45
	1	121 ± 44	ND	115 ± 24	117 ± 24	101 ± 49
CLO-BuOH	50	121 ± 23	100 ± 21	80 ± 6	104 ± 29	83 ± 37
	10	112 ± 32	ND	104 ± 14	109 ± 8	101 ± 44
	1	108 ± 24	ND	103 ± 24	133 ± 31	110 ± 50
CLO-EtOAc	50	113 ± 18	ND	90 ± 16	105 ± 14	101 ± 49
	10	122 ± 43	ND	111 ± 21	120 ± 12	104 ± 45
	1	101 ± 21	ND	110 ± 29	115 ± 25	100 ± 48
LPS	Ctr	100	100	100	100	100
Cell	Ctr	0	5 ± 4	2 ± 2	3 ± 3	0
DMSO	Ctr	0	5 ± 5	1 ± 1	4 ± 3	0
